# Emergency Department–Initiated Buprenorphine for Opioid Use Disorder: A Systematic Review of Treatment Engagement and Emergency Care Utilization

**DOI:** 10.7759/cureus.101792

**Published:** 2026-01-18

**Authors:** Mosab A Alabas, Abdulmajeed A Alshahrani, Saeed M Almobty, Ibrahim A Alaqeel, Mohammed S Alhomaidi, Mohammed S Alfuwis, Yasser A Alshammari, Laura M Damanhouri, Abdulmalek S Alyahya, Fawaz A Alshehri

**Affiliations:** 1 Department of Emergency Medicine, Aseer Central Hospital, Abha, SAU; 2 Department of Emergency Medicine, Armed Forces Hospital Southern Region, Khamis Mushait, SAU; 3 College of Medicine, University of Hail, Hail, SAU; 4 College of Medicine and Surgery, King Abdulaziz University, Jeddah, SAU; 5 College of Medicine, King Saud Bin Abdulaziz University for Health Sciences, Riyadh, SAU; 6 College of Medicine, King Abdulaziz University, Jeddah, SAU

**Keywords:** buprenorphine initiation, care transitions, emergency department, emergency department utilization, medication for opioid use disorder, opioid use disorder, substance use treatment, treatment engagement

## Abstract

Emergency departments (EDs) represent a critical point of contact for individuals with opioid use disorder (OUD). Initiation of buprenorphine, a medication for OUD, in the ED has been proposed as a strategy to improve engagement in ongoing treatment and reduce recurrent emergency care utilization; however, evidence across these outcomes has not been comprehensively synthesized. This systematic review aimed to qualitatively summarize the effectiveness of ED-initiated buprenorphine in improving treatment engagement and reducing subsequent ED utilization among adults with OUD. A systematic search of major biomedical databases identified randomized and observational studies evaluating buprenorphine initiation in the ED and reporting outcomes related to treatment engagement or return ED visits. Data were extracted using a standardized approach, and methodological quality was assessed using a validated appraisal tool. Due to heterogeneity in study designs and outcome measures, findings were synthesized qualitatively. Across included studies, ED-initiated buprenorphine was consistently associated with higher rates of engagement in OUD treatment within 30 days compared with referral or brief intervention alone. Evidence regarding reductions in subsequent ED utilization was mixed, with some studies demonstrating fewer opioid-related or all-cause ED return visits and others showing no significant differences, particularly over shorter follow-up periods. Social determinants of health, including housing stability, insurance status, and race, appeared to modify outcomes, and the presence of adjunctive ED-based services such as behavioral counseling and naloxone distribution was variably associated with improved engagement and reduced ED use. Overall, ED-initiated buprenorphine is an effective intervention for improving early treatment engagement among patients with OUD, while its impact on subsequent ED utilization appears context-dependent and influenced by social factors and the availability of supportive follow-up care.

## Introduction and background

Opioid use disorder (OUD) remains a major public health challenge, with persistently high rates of opioid-related morbidity and mortality despite the availability of effective treatments [1]. Emergency departments (EDs) serve as a key point of contact for individuals with OUD, particularly during episodes of overdose or acute withdrawal [2]. Many patients rely on EDs for episodic care and experience frequent repeat visits due to poor linkage to ongoing treatment, contributing to fragmented care and increased strain on emergency services [3]. Reducing recurrent ED utilization among this population is therefore an important clinical and health system priority [4].

Buprenorphine is an evidence-based medication for the treatment of OUD that reduces illicit opioid use, overdose risk, and mortality [5]. Initiating buprenorphine during acute ED encounters offers a critical opportunity to engage patients at a time of heightened vulnerability and potential readiness for change [6]. Although regulatory, logistical, and educational barriers historically limited buprenorphine use in emergency settings, evolving policies and growing clinical experience have increasingly supported the feasibility of ED-initiated buprenorphine as a bridge to longitudinal addiction care [7].

Existing randomized and observational studies demonstrate that ED-initiated buprenorphine improves short-term engagement in addiction treatment compared with referral or brief intervention alone [8]. However, its impact on downstream healthcare utilization, particularly repeat ED visits, is less well defined. Recurrent ED presentations among individuals with OUD are common and are associated with higher healthcare costs, disrupted continuity of care, and increased risk of adverse outcomes, making ED return visits a clinically meaningful outcome when evaluating ED-based interventions [9].

Despite expanding implementation of ED-initiated buprenorphine programs, evidence regarding their effect on short-term ED utilization remains heterogeneous. Reported outcomes vary based on patient characteristics, availability of follow-up care, and the presence of adjunctive services such as naloxone distribution or peer support. In addition, differences in study design, outcome definitions, and follow-up duration complicate the interpretation of the existing literature [10].

This systematic review aims to critically examine and qualitatively synthesize available evidence on ED-initiated buprenorphine among patients with OUD, with a focus on treatment engagement and short-term ED return visits. By consolidating current findings, this review seeks to inform emergency clinicians, health system leaders, and policymakers about the effectiveness of ED-initiated buprenorphine as a strategy to improve care continuity and reduce recurrent ED utilization.

## Review

Methodology

Literature Search Strategy

This systematic review was conducted in accordance with the Preferred Reporting Items for Systematic Reviews and Meta-Analyses (PRISMA) guidelines [11]. A comprehensive electronic search was performed in PubMed, Scopus, Web of Science, and the Cochrane Library from database inception through the final search date. The search strategy combined controlled vocabulary and free-text terms related to ED care, buprenorphine or buprenorphine/naloxone initiation, and opioid use disorder, along with terms capturing treatment engagement and ED utilization outcomes. The initial search was developed in PubMed and adapted for each database to account for differences in indexing and syntax. Searches were limited to human studies and original research articles. Reviews, editorials, commentaries, protocols without outcome data, conference abstracts without full results, and clearly non-relevant publications were excluded.

Eligibility Criteria

Eligibility criteria were defined a priori using a population-intervention-outcome framework. Studies were included if they evaluated adult patients with OUD treated in an ED and assessed buprenorphine initiation during the ED visit or at discharge. Eligible studies were required to report post-ED outcomes, including engagement in addiction treatment, repeat ED visits, or related follow-up measures within a defined time frame. Randomized controlled trials and observational studies, including prospective or retrospective cohort designs and chart reviews, were eligible. Studies conducted in any geographic or clinical setting were considered. Exclusion criteria included studies not involving ED-initiated buprenorphine, those limited to non-ED settings, studies focused solely on provider attitudes or implementation without patient-level outcomes, qualitative-only studies, case reports, narrative reviews, and non-English publications.

Study Selection

All retrieved records were imported into reference management software, and duplicates were removed. Study selection was performed in two stages. First, titles and abstracts were screened to exclude clearly irrelevant studies. Second, full-text articles of potentially eligible studies were reviewed against predefined inclusion and exclusion criteria. Reasons for exclusion at the full-text stage were documented, and the selection process was summarized using a flow diagram to ensure transparency and reproducibility.

Data Extraction and Quality Appraisal

Data were extracted using a standardized, pilot-tested form. Extracted variables included study design, setting, population characteristics, sample size, intervention details, comparator conditions, follow-up duration, and reported outcomes related to treatment engagement and ED utilization. Methodological quality was assessed using a modified version of the Downs and Black checklist [12,13], a validated tool for evaluating both randomized and nonrandomized studies of healthcare interventions. The modified checklist assesses study quality across key domains, including reporting quality, external validity, internal validity related to bias, internal validity related to confounding, and statistical power, with a maximum possible score of 27 points. Higher scores indicate better methodological quality. Quality assessments were used to inform interpretation of findings and to highlight methodological strengths and limitations across the included studies, rather than as criteria for study exclusion.

Results

Study Selection

**Figure 1:**
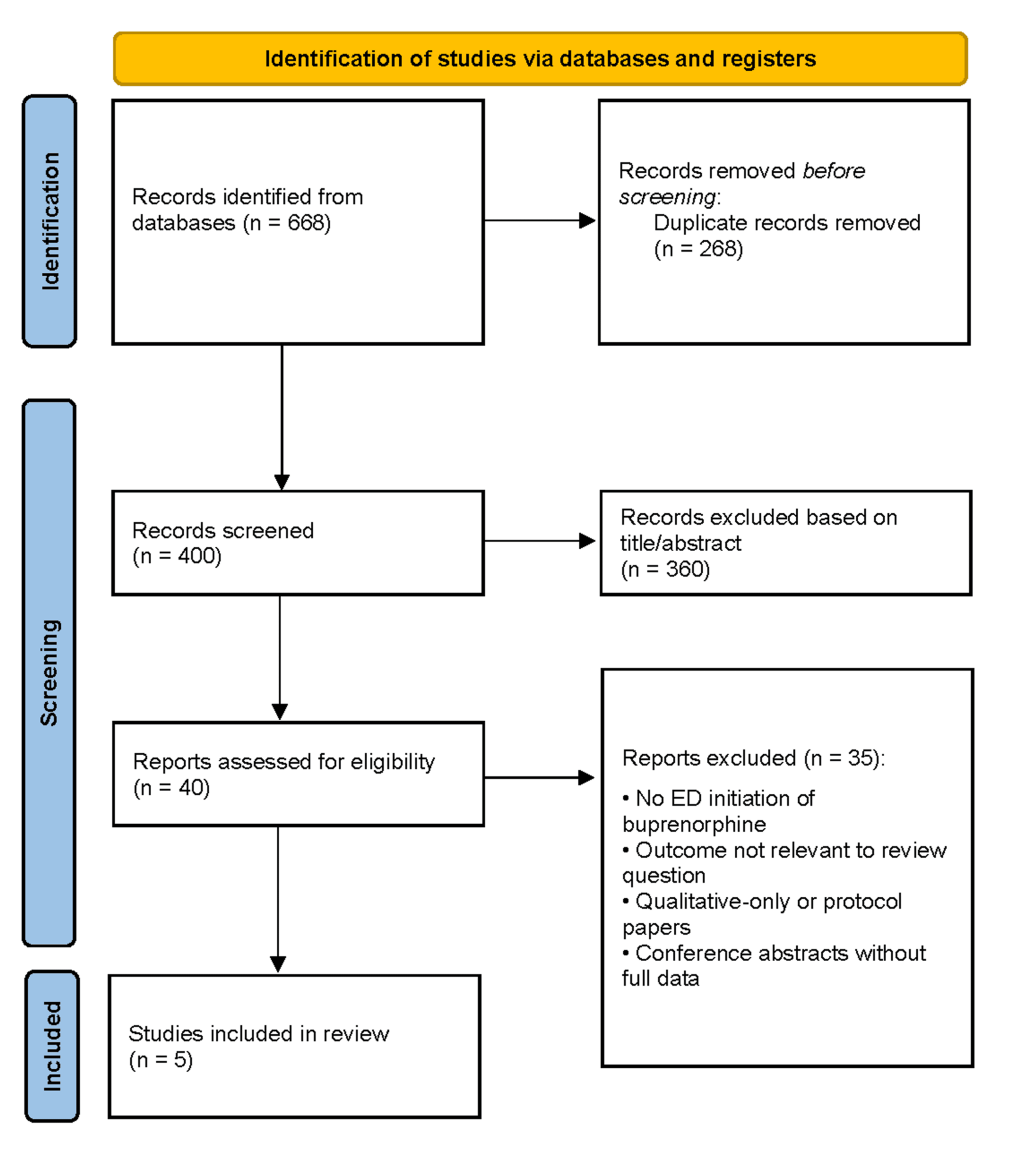
PRISMA flow diagram of study selection PRISMA flow diagram illustrating the identification, screening, eligibility assessment, and inclusion of studies in the systematic review. The diagram details the number of records identified through database searching and other sources, records screened after removal of duplicates, full-text articles assessed for eligibility, and studies included in the final qualitative and/or quantitative synthesis, with reasons for exclusion at each stage.

The database search yielded 668 records. After removal of duplicates, 400 unique records underwent title and abstract screening, of which 360 were excluded for lack of relevance, inappropriate study design, or absence of relevant outcomes. Forty full-text articles were reviewed in detail, and 35 were excluded due to lack of ED-initiated buprenorphine, absence of treatment engagement or utilization outcomes, qualitative-only designs, or insufficient data. Five studies met the inclusion criteria and were included in the qualitative synthesis and quality assessment (Figure 1) [1-5].

Study Characteristics

All included studies were conducted in the United States and comprised one randomized controlled trial and four observational studies, including retrospective cohort studies and chart reviews. Study settings ranged from single urban academic EDs to multisite and nationwide claims-based analyses. Adult patients presenting with opioid overdose, dependence, or withdrawal were included, with sample sizes ranging from 169 to 6,451 participants. Most studies focused on patients not engaged in treatment at the index ED visit and discharged home. Follow-up periods ranged from 30 to 90 days, with one study assessing ED utilization outcomes up to one year. Interventions included ED-administered buprenorphine, buprenorphine or buprenorphine/naloxone prescriptions at discharge, and facilitated linkage to outpatient care. Comparators included referral-only approaches, brief interventions, or no comparator in descriptive cohorts. Primary outcomes were treatment engagement and repeat ED utilization (Table 1).

**Table 1:** Characteristics of the studies included in the systematic review Summary of included studies evaluating emergency department (ED)–initiated buprenorphine for opioid use disorder (OUD), describing study design, setting, population, interventions, comparators, follow-up duration, and outcomes related to treatment engagement and ED utilization. ED-initiated buprenorphine includes medication administered during the ED visit and/or prescribed at discharge, with or without facilitated referral to outpatient care. Primary outcomes were categorized as treatment engagement (initiation or receipt of formal addiction treatment within the specified follow-up period) or ED utilization (repeat ED visits). Abbreviations: ED: emergency department; OUD: opioid use disorder; MOUD: medications for opioid use disorder; MAT: medication-assisted treatment; aOR: adjusted odds ratio; ARD: adjusted risk difference; IRR: incidence rate ratio; CI: confidence interval; DSM-IV: Diagnostic and Statistical Manual of Mental Disorders, Fourth Edition; ICD-10: International Classification of Diseases, Tenth Revision; USA: United States of America

Study ID	Country	Study design	Setting (ED type / sites)	Population	Sample size	Inclusion criteria	Intervention (ED-initiated buprenorphine details)	Comparator/control	Follow-up duration	Primary outcome	Outcome type	30-day ED return visits (Yes/No)	30-day ED return visit data	30-day treatment engagement (Yes/No)	30-day treatment engagement data	Key quantitative results	Statistical analysis	Adjustments/confounders	Main conclusions	Limitations
Chambers et al. [1]	USA	Retrospective cohort	Four EDs in Rhode Island	Adults treated in the ED for opioid overdose, not engaged in OUD treatment, were discharged home	1,008	ED-treated opioid overdose; not in OUD treatment at index visit; discharged home	Buprenorphine initiated in or from the ED (administered in ED or prescribed at discharge)	No buprenorphine initiation / other ED services	30 days	Engagement in OUD treatment	Treatment engagement	No	Not reported	Yes	146/1,008 (14%)	ED buprenorphine initiation associated with higher 30-day engagement (aOR 5.86; 95% CI 2.70–12.71)	Multivariable conditional logistic regression	Age, sex, race/ethnicity, insurance, calendar year, prior overdose, ED site	ED initiation is strongly associated with increased treatment engagement.	Observational design; low buprenorphine initiation rate; ED utilization not assessed
Kilaru et al. [2]	USA	Retrospective cohort	Nationwide EDs (claims-based)	Adults treated in the ED for nonfatal opioid overdose, discharged home	6,451	ED visit for nonfatal opioid overdose; commercially insured; discharged home	Post-ED receipt of MOUD (buprenorphine or naltrexone); ED initiation not directly measured	No MOUD receipt	Up to 90 days	Receipt of OUD treatment	Treatment engagement	No	Not reported	Yes	16.6% received OUD treatment	MOUD receipt uncommon; Black patients had a lower adjusted probability (ARD −5.9%)	Multivariable logistic regression with predictive margins	Age, sex, race/ethnicity, region, overdose type, prior mental health treatment	Engagement after overdose is low, with demographic disparities.	ED initiation not directly measured; limited to commercially insured patients
Skains et al. [3]	USA	Retrospective chart review	Single urban academic ED	Adults discharged from the ED with a primary OUD diagnosis	169	ICD-10 OUD diagnosis; discharged from ED	Buprenorphine/naloxone prescribed at discharge (53.8%); often co-prescribed with naloxone	No buprenorphine prescription	30 days, 90 days, 1 year	Repeat ED utilization	ED utilization	Yes	Opioid-related ED visits at 30 days lower (37.5% vs 62.5%, P=0.04); adjusted IRR 0.56 (95% CI 0.33–0.96)	No	Not reported	Reduced ED utilization at 30 days, sustained to 1 year	Bivariate analyses; Poisson regression	Age, domicile status	ED-initiated buprenorphine reduces repeat ED utilization.	Single center; outside-system visits not captured
D’Onofrio et al. [4]	USA	Randomized controlled trial	Single urban academic ED	Adults with opioid dependence presenting to the ED	329	DSM-IV opioid dependence; not in treatment; medically stable	ED-initiated buprenorphine/naloxone with referral	Referral alone; brief intervention + referral	30 days	Engagement in addiction treatment	Treatment engagement	Yes	No significant difference (P = .51)	Yes	78% vs. 37% vs 45%	Significantly higher engagement and reduced illicit opioid use	χ² tests; mixed-model analyses	Randomization stratified by sex, cocaine use, opioid type	ED-initiated buprenorphine improves short-term engagement.	Single site; short follow-up; bundled intervention
Childers et al. [5]	USA	Observational descriptive cohort	Single urban academic ED (California Bridge)	Adults with OUD identified in the ED	210	OUD; accepted referral for outpatient MAT	Buprenorphine in ED (80.8%); discharge prescription (67.6%); care navigation	No comparator	30 days	Engagement in addiction treatment	Treatment engagement	No	Not reported	Yes	95/210 (45.2%)	Housing status predicted engagement (aOR 2.49; 95% CI 1.20–5.20)	Univariable and multivariable logistic regression	Age, sex, race/ethnicity, housing, mental health, insurance	High engagement despite high homelessness.	No comparator; single center

Quality Assessment

Methodological quality, assessed using the Modified Downs and Black checklist, ranged from good to high across studies, with total scores between 20 and 25 out of a possible 27. Reporting quality was consistently strong, and external validity was moderate to high. Internal validity related to bias and confounding was generally robust, particularly in the randomized trial [4], while observational studies demonstrated appropriate adjustment strategies. All studies met criteria for sufficient statistical power (Table 2).

**Table 2:** Methodological quality assessment of the included studies Quality assessment of the included studies based on the Downs and Black methodological checklist [13].

Study ID	Reporting (10)	External validity (3)	Internal validity – bias (7)	Internal validity – confounding (6)	Power (1)	Total score (27)	Overall quality
Chambers et al. [1]	9	3	6	3	1	22	Good
Kilaru et al. [2]	8	2	5	4	1	20	Good
Skains et al. [3]	8	2	6	4	1	21	Good
D’Onofrio et al. [4]	9	2	7	6	1	25	High
Childers et al. [5]	8	2	5	4	1	20	Good

Qualitative Synthesis

Across studies, ED-initiated buprenorphine was consistently associated with improved short-term engagement in OUD treatment. In the randomized trial, treatment engagement at 30 days was substantially higher among patients receiving ED-initiated buprenorphine compared with referral or brief intervention alone [4]. Observational studies reported similar trends, with buprenorphine initiation in or from the ED independently associated with higher odds of treatment engagement within 30 days [1,5]. Follow-up treatment rates varied across cohorts but were consistently higher among patients receiving buprenorphine during the ED encounter or at discharge [1,2,5].

Evidence regarding ED utilization outcomes was mixed. The randomized trial found no significant differences in ED use at 30 days between intervention groups [4]. By contrast, one observational study reported reduced opioid-related ED return visits at 30 days among patients prescribed buprenorphine, with adjusted analyses demonstrating reductions in all-cause ED visits at 30 days, 90 days, and one year [3]. Non-opioid-related ED visits appeared to be more strongly associated with demographic and social factors than with buprenorphine exposure [3].

Several studies evaluated the influence of adjunctive ED-based services. Behavioral counseling in the ED was associated with increased treatment engagement in one study, whereas referral alone and naloxone distribution were not [1]. Naloxone provision was associated with reduced opioid-related ED return visits in another study, independent of buprenorphine prescription [3]. Social determinants, including housing instability, insurance status, race, age, and sex, were consistently associated with disparities in treatment engagement and follow-up across studies, even after adjustment for clinical factors [1,2,5].

Limitations

This review has several limitations. The small number of eligible studies and substantial heterogeneity in study design, outcome definitions, and follow-up durations limited the ability to perform a quantitative meta-analysis and constrained direct comparisons across studies. Most included studies were conducted in academic EDs in the United States, which may limit generalizability to nonacademic, rural, or international settings. Variation in intervention components, comparator conditions, and availability of follow-up services further complicates the interpretation of observed effects. In addition, many studies were observational in nature and therefore subject to residual confounding and selection bias. Future research should prioritize standardized outcome measures, longer follow-up periods, and comparative evaluation of different ED buprenorphine delivery models, including extended-release formulations and structured warm-handoff programs. Rigorous assessment of equity-focused interventions is also needed to ensure that the benefits of ED-initiated buprenorphine are realized across diverse patient populations.

## Conclusions

This systematic review demonstrates that ED-initiated buprenorphine is consistently associated with improved short-term engagement in treatment for OUD, particularly within the first 30 days after the index ED visit. Initiating buprenorphine in or from the ED appears more effective than referral-based or nonpharmacologic approaches alone in facilitating linkage to ongoing addiction care. By contrast, the effect of ED-initiated buprenorphine on subsequent ED utilization is less consistent and varies according to follow-up duration, outcome definitions, accompanying interventions, and social determinants of health. While some studies report reductions in opioid-related or all-cause return visits, these findings are not uniform across settings or time frames. Overall, the evidence supports ED-initiated buprenorphine as a key component of evidence-based care for OUD, while underscoring the importance of integrated care models that combine pharmacologic treatment with behavioral support, harm reduction strategies, and reliable outpatient follow-up to achieve sustained improvements in patient and health system outcomes.
